# Identification and characterization of long non-coding RNAs in subcutaneous adipose tissue from castrated and intact full-sib pair Huainan male pigs

**DOI:** 10.1186/s12864-017-3907-z

**Published:** 2017-07-19

**Authors:** Jing Wang, Liushuai Hua, Junfeng Chen, Jiaqing Zhang, Xianxiao Bai, Binwen Gao, Congjun Li, Zhihai Shi, Weidong Sheng, Yuan Gao, Baosong Xing

**Affiliations:** 1 Henan Key Laboratory of Farm Animal Breeding and Nutritional Regulation, Institute of Animal Husbandry and Veterinary Science, Henan Academy of Agricultural Sciences, No.116 Huayuan road, Zhengzhou, 450002 People’s Republic of China; 2 United States Department of Agriculture-Agricultural Research Service, Bovine Functional Genomics Laboratory, Beltsville, MD 20705 USA; 3 Xinxian Bureau of Animal Husbandry, Xinxian, 465550 Beijing, People’s Republic of China

**Keywords:** Subcutaneous adipose tissue, LncRNA, Pig, Fat deposition

## Abstract

**Background:**

Long non-coding RNAs (lncRNAs) regulate adipose tissue metabolism, however, their function on testosterone deficiency related obesity in humans is less understood. For this research, intact and castrated male pigs are the best model animal because of their similar proportional organ sizes, cardiovascular systems and metabolic features.

**Results:**

We identified lncRNAs in subcutaneous adipose tissue by deep RNA-sequencing using the intact and castrated Huainan male pigs. The results showed that castration reduced serum testosterone but increased body fatness-related traits (serum triglyceride levels, backfat thickness, intramuscular fat content, and adipocyte size). Meanwhile, 343 lncRNAs from subcutaneous adipose tissue were identified, including 223 intergenic lncRNAs (lincRNAs), 68 anti-sense lncRNAs, and 52 intronic lncRNAs. It was predicted that there were 416 recognition sites for C/EBPα in the 303 lncRNA promoter region, and 13 adipogenesis-promoting miRNAs and five adipogenesis-depressing miRNAs target these lncRNAs. Eighteen lncRNAs, including nine up- and nine down-regulated had more than 2-fold differential expression between the castrated and intact male pigs (*q-*value < 0.05). Functional analysis indicated that these 18 lncRNAs and their target genes were involved in fatty acid, insulin, and the adipocytokine signaling pathway. We further analyzed the features of a conserved mouse lncRNA gene ENSMUST00000189966 and found it mainly expressed in the cell nucleus and target the Nuclear Receptor Subfamily 2 Group F Member 2 (*NR2F2*) gene. In 3 T3-L1 cells, differentiation down-regulated their expression, but dihydrotestosterone (DHT) significantly up-regulated their expression in a concentration-dependent manner (*P* < 0.05).

**Conclusions:**

These results suggested that lncRNAs and their target genes might participated in the castration-induced fat deposition and provide a new therapeutic target for combatting testosterone deficiency-related obesity.

**Electronic supplementary material:**

The online version of this article (doi:10.1186/s12864-017-3907-z) contains supplementary material, which is available to authorized users.

## Background

Sex glands not only play a regulatory role in reproductive traits, but also in growth traits [1]. Researchers have found that gonadal steroid hormones like testosterone could promote lipolysis and releasing energy. In the process of aging or illness, reduced levels of androgens are associated with obesity (particularly in visceral fat) and related diseases in human [2]. The castrated male pigs, with the similar organ size, metabolic features, cardiovascular systems, are the best animal model for researching human testosterone deficiency-related diseases [3–5].

Long noncoding RNAs (lncRNAs) are transcripts that structurally resemble mRNAs but do not encode proteins. LncRNAs regulate gene expression at the post-transcriptional level and play important roles in numerous disease and physiological metabolism processes, including X-chromosome inactivation [6], embryonic development [7], pluripotency maintenance [8]. Recently, a growing number of reports has demonstrated that lncRNAs regulated adipogenesis by activating or silencing key genes through recruiting chromatin modification complexes or acting as competing endogenous RNAs (ceRNAs): ADINR (adipogenic differentiation induced noncoding RNA), promoted adipogenesis by activating CCAAT/enhancer binding protein alpha (C/EBPα) transcription in *cis* [9]. LncRNA-AK038898 (Blnc1) promoted brown adipocyte differentiation and stimulated the thermogenic phenotype by forming a complex and feedforward regulatory loop with transcription factor EBF2 [10]. PU.1 (also known as SPI1, spleen focus forming virus (SFFV) proviral integration oncogene) inhibited adipogenesis by the down-regulation of adipogenic master gene PPARγ [11], and its antisense long non-coding RNAs promotes adipogenesis by preventing its mRNA translation [12]. LncRNA ADNCR (adipocyte differentiation-associated long noncoding RNA) suppressed adipocyte differentiation by function as a ceRNA for miR-204, whose target gene-SIRT1 was known to repress PPARγ activity and inhibit adipocyte differentiation [13].

The regulatory function of lncRNA on fat metabolism suggests that it is feasible to research the testosterone deficiency-related fat deposition from the angle of lncRNA. Therefore, we investigated the changes of lncRNA expression in the subcutaneous adipose tissue (SAD) between intact and castrated male pigs, and this provides a novel view of the role of lncRNAs in testosterone deficiency-induced fat deposition as well as basic prophylactic-therapeutic measures for human sex hormone deficiency-related obesity.

## Results

### Body fatness traits

In this research, castrated and intact male pigs had similar birth, weaning, and carcass weights (*P* > 0.05). However, the leaf fat weight (nine times greater, *P* = 0.007), fat percentage (*P* = 0.020), and 6–7th rib fat (five times thicker, *P* = 0.001) in castrated male pigs were significantly different with that in intact male pigs (Table 1). Castration resulted in a significant increase in intramuscular fat content in male pigs’ longissimus dorsi muscle (LDM, *P* < 0.01) and psoas major muscle (PMM, *P* < 0.05, Table 1).

**Table 1:** The effect of castration on body fatness traits and serum items in male pigs

	Intact pigs (*n* = 3, Mean ± s.e.m.)	Castrated pigs (*n* = 3, Mean ± s.e.m)	*P*-value
Body fatness traits			
Birth weight (kg)	1.283 ± 0.109	1.217 ± 0.085	0.550
Body weight (kg)	130.000 ± 7.550	125.667 ± 4.841	0.274
Carcass weight (kg)	96.500 ± 3.761	94.266 ± 0.545	0.763
Leaf fat weight (kg)	0.177 ± 0.026	1.657 ± 0.131	0.007
Fat percentage (%)	7.700 ± 0. 700	26.000 ± 2.100	0.020
6–7th rib fat thickness (mm)	10.313 ± 0.515	55.407 ± 1.241	0.001
IMF of the LDM (%)	4.500 ± 0.300	5.830 ± 0.500	0.007
IMF of the PMM (%)	4.300 ± 0.600	5.240 ± 0.900	0.032
Serum items			
Testosterone (ng/mL)	11.231 ± 0.387	0.159 ± 0.016	0.001
Triglyceride (mM)	0.217 ± 0.006	0.319 ± 0.003	0.008
Total cholesterol (mM)	0.673 ± 0.035	1.052 ± 0.052	0.034

*IMF* intramuscular fat content

**Fig. 1:**
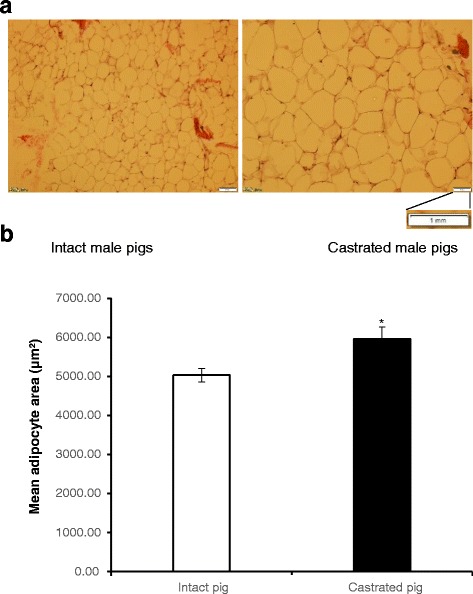
Histological sections of subcutaneous adipose tissue from intact and castrated male pigs. **a** H&E staining of subcutaneous adipose tissue from intact and castrated male pigs (shown at ×200 magnification). **b** The mean adipocyte size in intact and castrated male pigs. The adipocyte area was measured using Image J software from three different animals per group. *P < 0.05

Castration significantly reduced the serum testosterone level (*P* < 0.01). However, the serum triglyceride (TG) was up-regulated by castration (*P* < 0.01, Table 1). These results corresponded to the larger adipocyte size (*P* < 0.01) observed in castrated group compared with intact group (Fig. 1).

### Read mapping and transcript assembly

The redundant and low-quality reads were removed, and a total of 11.60 G and 10.84 G clean bases were obtained in castrated and intact male pigs, respectively. The clean reads were aligned to the reference genome for assembly. Approximately, the mapping rate of the total clean reads to the *Sus scrofa* genome assembly 10.2 were 81.31% (castrated male pigs) and 82.48% (intact male pigs), respectively.

### Genomic information of porcine lncRNAs

In this research, 343 lncRNA genes were found distributed in all chromosomes (Additional file 1: Text file containing identified lncRNA sequence), including 223 intergenic lncRNAs (lincRNAs), 68 anti-sense lncRNAs, and 52 intronic lncRNAs. These 343 lncRNA genes corresponding to 402 transcripts, about 1.2 isoforms per lncRNA locus on average. The size of porcine lncRNAs ranged from 203 to 21,530 nucleotides, the average size was approximately 1520 bp. The average exon number of these lncRNA was 2.1 on average (90.1% of the total lncRNAs has two exons), the same as that of human lncRNAs [14]. The average length of lncRNAs open reading frame (ORF) was 110 bp. We found that 73.5% of the total lncRNA had target genes (protein-coding genes located in 100 kb around the lncRNAs), containing 3.4 isoforms per lncRNA locus on average, respectively.

In this research the mean fragments per kilobase of exons per million fragments mapped (FPKM) value of these lncRNAs was 17, and 91.2% of the lncRNAs with FPKM values less than 15 [15], indicating that the expression levels of the majority of porcine lncRNAs were low, which was similar to the expression levels of human lncRNAs [16]. In this research, we found 562 recognition sites for C/EBPα in the 300 identified lncRNAs promoter region, containing 1.9 C/EBPα recognition site per lncRNA on average (Additional file 2: Table S1). The relationship between the 343 lncRNAs with 18 adipogenesis-related miRNAs was shown in Table S2 (Additional file 3). We found 13 adipogenesis-promoting miRNAs (let-7、miR-9、miR-15a、miR-17、miR-21、miR-24、miR-30、miR-103、miR-107、miR-125b、miR-204、miR-210、and miR-378) target 860 lncRNA loci. Five depressing-adipogenesis miRNAs (miR-27, miR-150, miR-221, miR-222, and miR-326) target 217 lncRNAs.

### The difference between lncRNAs with protein-coding genes

We also obtained 12,372 protein-coding genes, with 1.2 isoforms on average (38,219 transcripts in total). These protein-coding genes had 518 bp ORF length and 9.8 exons on average, larger than that of the lncRNA genes (Additional file 4: Figure S1a, b). There was only 6.24% of protein-coding transcripts with two exons, far less than that of lncRNA genes (Additional file 4: Figure S1c).

### Differentially expressed lncRNAs in SAD between castrated and intact male pigs

**Fig. 2:**
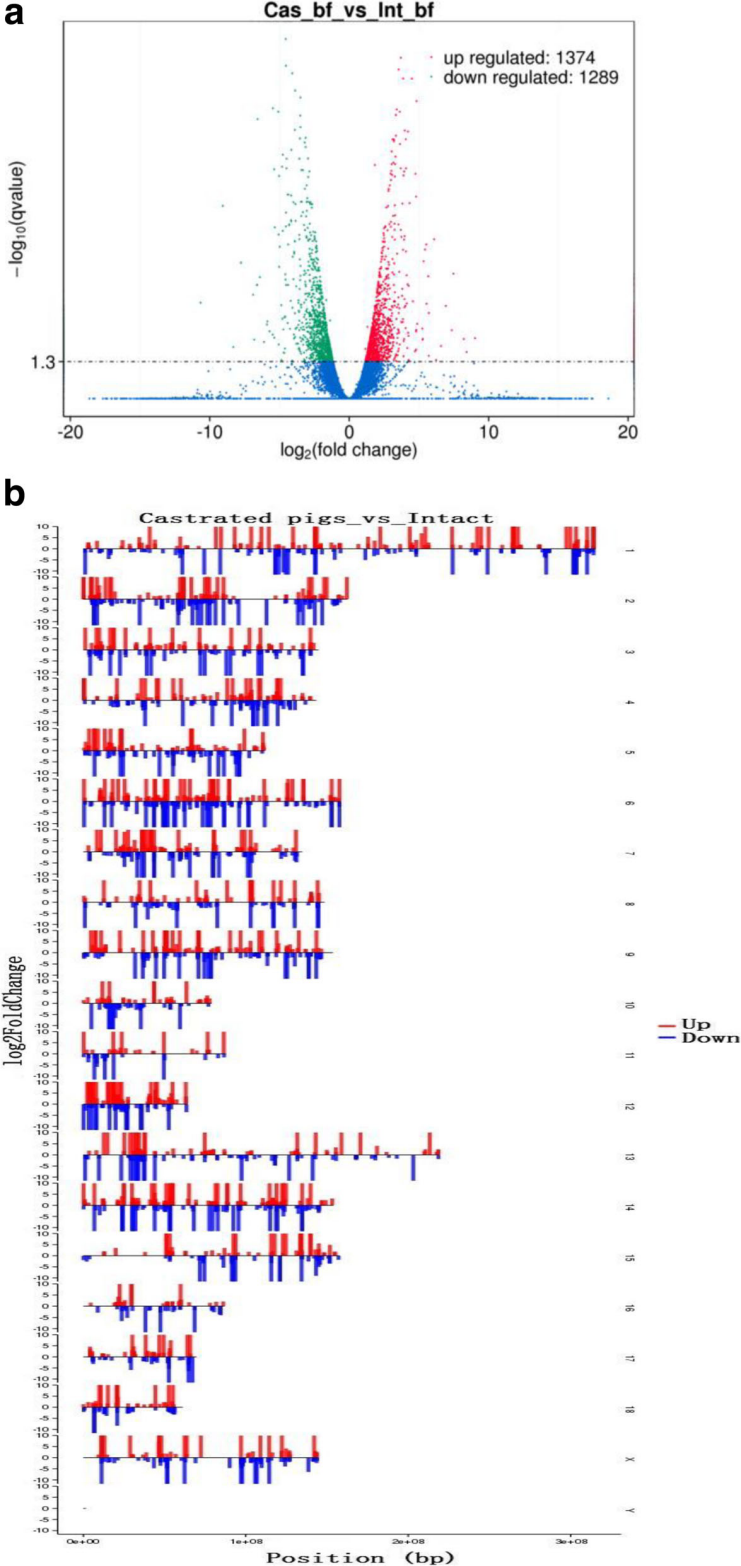
The differentially expressed lncRNAs and mRNA between castrated and intact male pigs and their distribution. **a** The differentially expressed lncRNAs and mRNA between castrated and intact male pigs. Each point in the figure represents a lncRNA or mRNA. The X axis show the position of lncRNAs and mRNA in different chromosomes, the Y axis show the log_2_FoldChange. The *red points* represent up-regulated lncRNAs or mRNA. The *green points* represent down-regulated lncRNAs or mRNA. The blue points represent equally expressed lncRNAs or mRNA. **b** The distribution of the differentially expressed lncRNAs and mRNA. The Y axis show log_2_normalized read counts. The red bars represent up-regulated lncRNAs or mRNA. The green bars represent down-regulated lncRNAs or mRNA

Eighteen lncRNAs (including nine up- and nine down-regulated, Additional file 5: Table S3) and 2645 mRNA (including 1365 up- and 1280 down-regulated, Additional file 6: Table S4), were found to differentially express in SAD between the castrated and intact male pigs (*q*-value < 0.05, Fig. 2a). The chromosome distribution shows these mRNAs and lncRNAs location in all of the chromosomes (Additional file 7: Figure S2b).

To validate the sequencing data, eight lncRNAs (four up- and four down-regulated) were randomly selected, and their expression were confirmed via the qRT-PCR method. We calculated the correlation efficiency of qRT-PCR and RNA-sequencing data using SPSS software (version 1.70), and the result supported our sequence data (*R* = 0.882, Additional file 7: Figure S2).

### KEGG analysis of the target genes of 18 lncRNAs and the differently expressed protein-coding genes

**Fig. 3:**
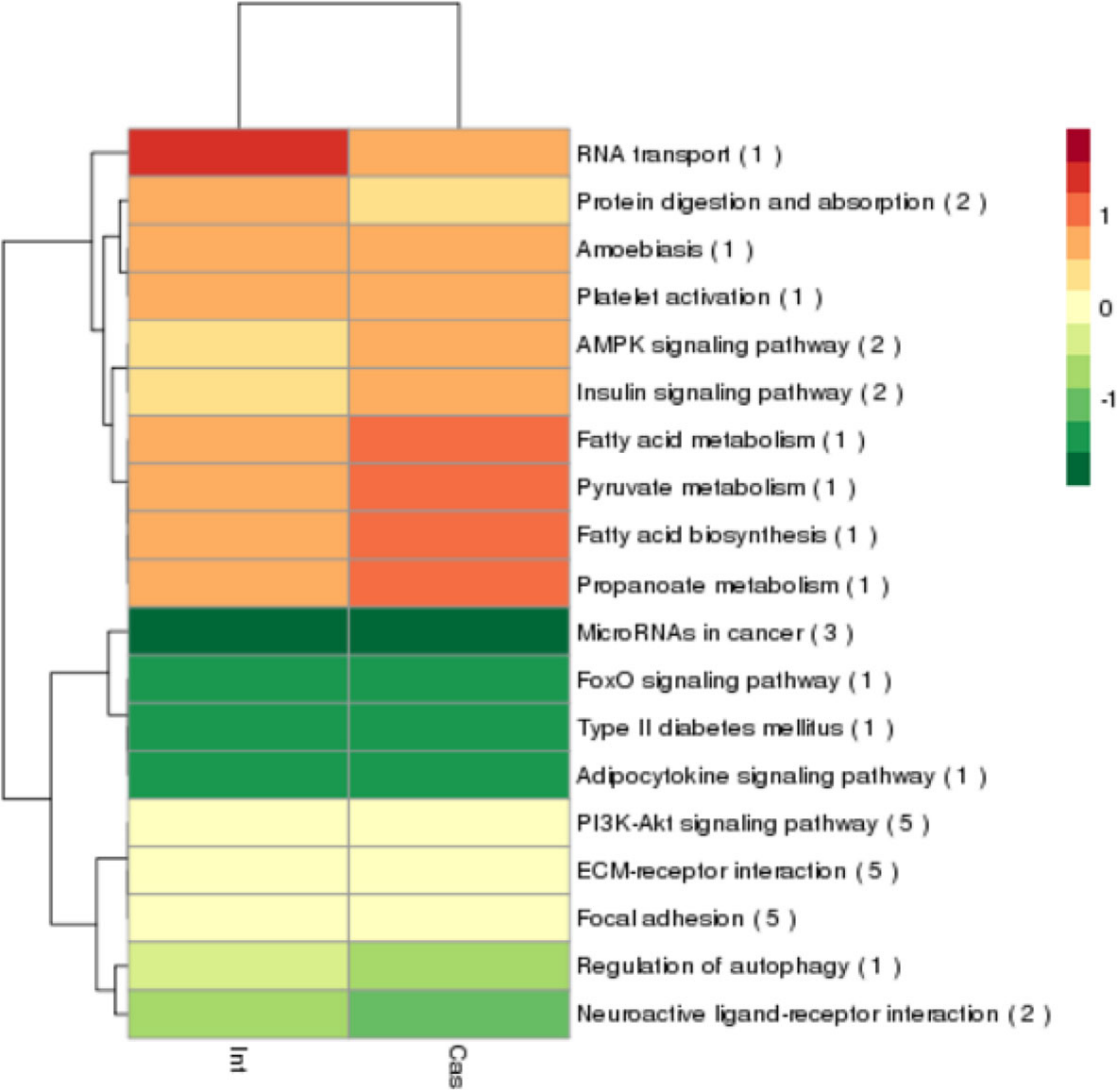
KEGG pathways enriched for the closely neighboring protein-coding genes of differently expressional lncRNAs in the subcutaneous adipose of castrated and intact male pigs. *Green* and *red* represent lower-than-average and higher-than-average signal levels, respectively. The numbers behind the pathway name represent the numbers of differentially expressed genes in that particular pathway. Int: intact male pig, Cas: castrated male pig

**Fig. 4:**
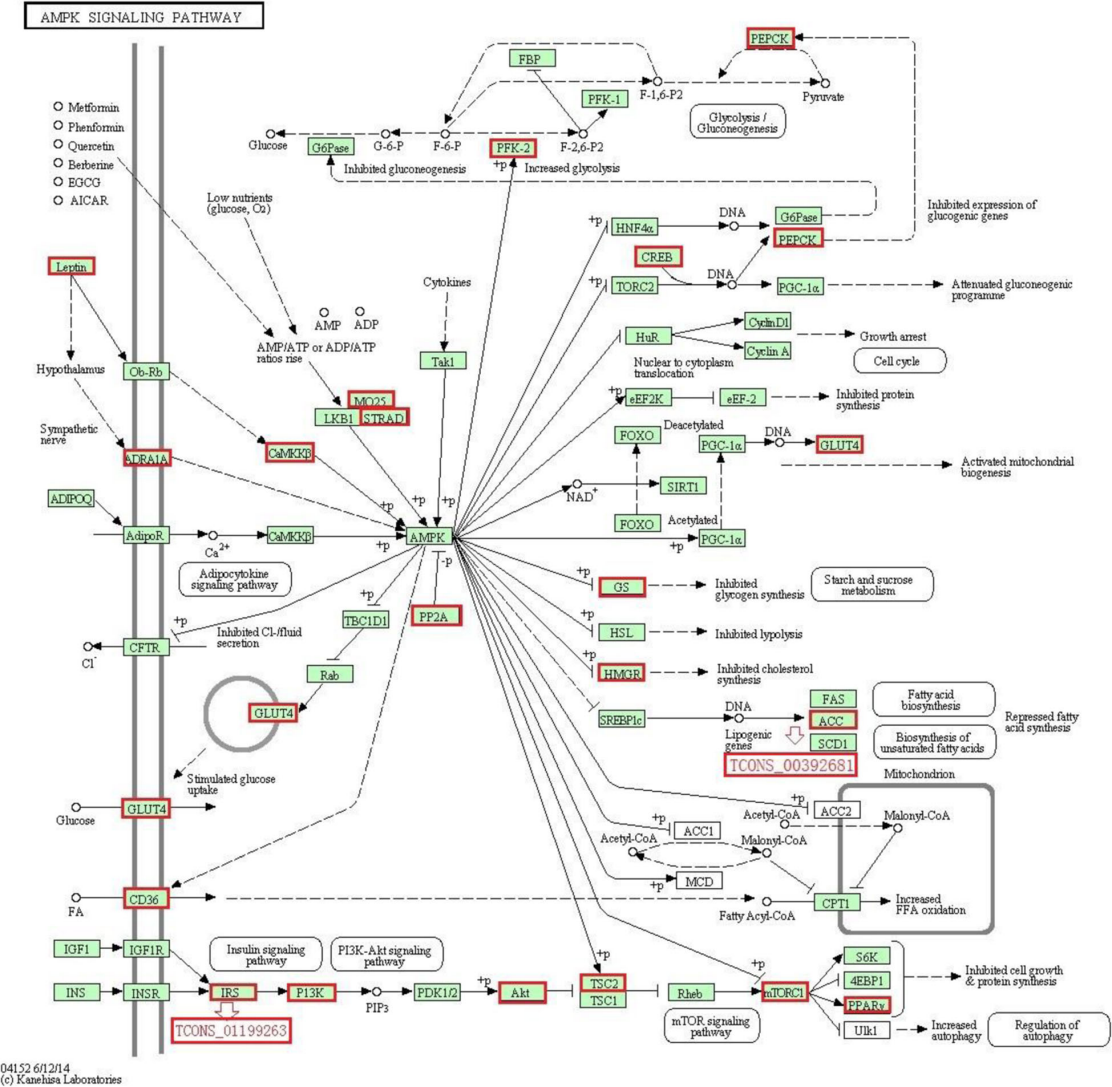
Differently expressed protein-coding genes and lncRNAs participating in the AMPK signaling pathway via DAVID KEGG analysis. The *red boxes* represent the upregulated protein-coding genes and lncRNAs, the *red hollow arrow* showed the target relationship between lncRNAs and their predicted target genes

According to KEGG functional annotations, the predicted target genes of nine up- and nine down-regulated lncRNAs were further classified to identify pathways, and 19 pathways are shown in Fig. 3. It was worth noting that the up-regulated target genes of the majority of lncRNAs in the castrated pigs belonged to the adipocytokine, insulin, fatty acid metabolism, and fatty acid biosynthesis signaling pathways, which were closely related to lipogenesis [17–19]. Another pathway targeted by up-regulated protein-coding genes and lncRNAs is the AMPK signaling pathway (Fig. 4), which is known to be involved in increasing the adipocyte volume and number [20]. Acetyl-CoA carboxylase beta (ACACA) and insulin receptor substrate 1 (IRS1) were the predicted target genes for lncRNAs TCONS_00392681 and TCONS_01199263, respectively. They all upregulated by castration, which indicated that these protein-coding genes and lncRNAs participated in the castration induced lipid deposition by increasing the adipocyte volume and number.

### Characterization of ENSMUST00000189966

Nuclear Receptor Subfamily 2 Group F Member 2 (NR2F2, also known as COUP-TF II) played an important role in the regulation of both hormone and lipid [21]. So TCONS_01646544, one of the downregulated lncRNAs and target NR2F2, was chosen to validate the identified lncRNAs’ response to dihydrotestosterone (DHT) and differentiation. To raise efficiency, ENSMUST00000189966, a TCONS_01646544 homologous in mice, was used in further research with the 3 T3-L1 cell line.

**Fig. 5:**
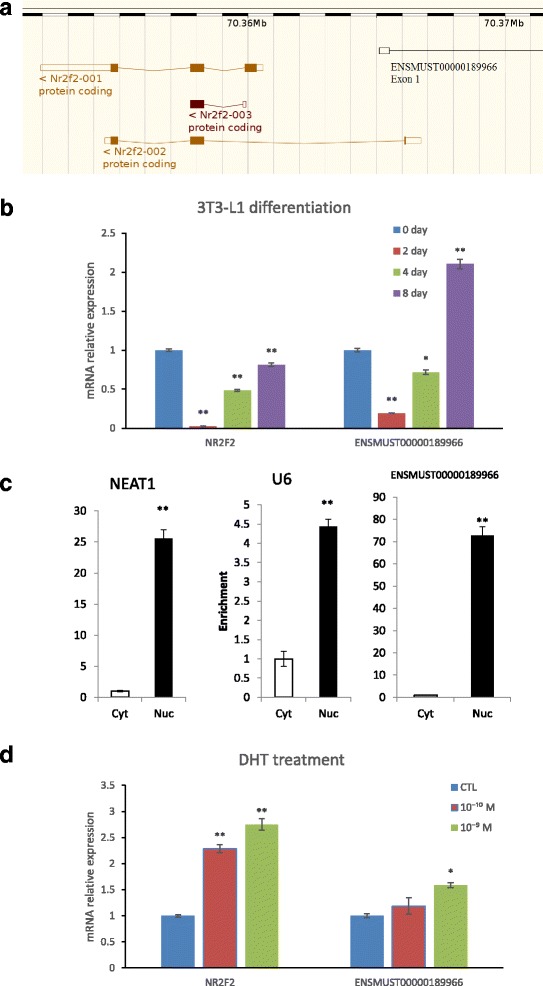
Features of NR2F2 and ENSMUST00000189966. **a** Gene structure of NR2F2 and ENSMUST00000189966. NR2F2–001, NR2F2–002 and NR2F2–003 are different isoforms. The scale in the top show the location in the chromosome 7. ENSMUST00000189966 exon 1 overlapped with NR2F2–002 intron 2. **b** Q-PCR analysis of NR2F2 and ENSMUST00000189966 gene expression during 3 T3-L1 cell culture in the differentiation medium (DM) for 0, 2, 4, and 8 days. **c** qPCR analyses of cytosolic (Cyt) and nuclear (Nuc) fractions. **d** The effect of dihydrotestosterone (DHT) (control (CTL), 10^−9^, and 10^−10^ M) on NR2F2 and ENSMUST00000189966 gene expression in 3 T3-L1 cells. The values represent the means ± s.e.m., *n* = 3. **indicates *P* < 0.01, *indicates *P* < 0.05

ENSMUST00000189966 and *NR2F2* reached a minimum expressional level on day 2 of differentiation and then gradually rose. On day 8, the *NR2F2* expression level was still lower than that on day 0; however, the expression of ENSMUST00000189966 on day 8 was much higher than that on day 0 (*P* < 0.01, Fig. 5a). We found that ENSMUST00000189966 was mainly expressed in nucleus of the 3 T3-L1 cells (Fig. 5b).

On day 14 of differentiation, Oil-red O staining was used to confirm the maturity of the 3 T3-L1 cells, then DHT treatment was started. DHT significantly up-regulated ENSMUST00000189966 (*P* < 0.05) and *NR2F2* (*P* < 0.01) expression in a concentration-dependent manner (Fig. 5c).

## Discussion

In humans, testosterone deficiency causes obesity and associated diseases [15]. Researchers have identified its molecular mechanism from genome and transcriptome angles, using castrated male pigs as the model animal [22, 23]. However, it remains unclear whether testosterone deficiency can influence lncRNA expression patterns in adipose tissue. In this research, we first combined body fatness traits, serum testosterone and TG levels, and histochemistry analysis with lncRNA sequencing to clarify the molecular mechanism underlying the increased body fatness response to testosterone deficiency.

Several recent studies reported that lncRNAs were directly regulated by key transcription factors that drive cellular differentiation [24, 25]. In this research, we found 562 recognition sites for C/EBPα in the 300 lncRNA promoter region. Sun et al. (2013) also found that *PPARγ* and *C/EBPα* were physically bound within the promoter region of adipose-enriched lncRNAs [26]. These indicated that lncRNA play an important role in lipid metabolism by interaction with key transcription factors.

Cai et al. (2014) found that 18 miRNAs were differentially expressed between intact and castrated male pigs, including miR-15a, miR-21, miR-27, miR-30, and so on [23]; Bai et al. (2014) reported that 177 miRNAs had more than 2-fold differential expression between castrated and intact male pigs, including miR-21, miR-30, miR-27, miR-103, and so on [22]. Our results were consisted with these reports, it was predicted that there were lncRNAs were the target genes for miR-21, miR-30, and miR-27. These indicated that miR-21, miR-30, and miR-27 and their target lncRNAs may play an important role in the androgen deficiency-related fat deposition, as it is widely known that miR-30a targets the androgen receptor (AR) gene [22].

The significant change after castration was the reduction of androgens, so it was predicted that *AR* plays an important role in androgen-deficiency related fat deposition. However, there is dissension regarding the effect of castration on the *AR* expression level: Bai et al. (2014) reported that *AR* expression in porcine backfat dramatically decreased after castration [22]; Choi et al. (2010) reported that the expressional levels of the *AR* gene in LD muscle were higher in bulls than steers, but in fat tissue its expression did not differ between bulls and steers [27]. Zhou et al. (2014) found that no significant difference in the *AR* expressional level of adipose tissues between bulls and steers (*P* > 0.05). In this research, we found that castration significantly down-regulated the *AR* level in SAD (*P* < 0.05). The effect of *AR* on testosterone deficiency-related fat deposition needs to be further research, but we have not found lncRNA around the porcine *AR* gene.

It was notable that ENSMUST00000189966 and its target gene *NR2F2* were significantly down-regulated after castration (*P* < 0.01). The *NR2F2* gene encodes a member of the steroid thyroid hormone superfamily of nuclear receptors, and it was widely known that sex hormones belong to the steroid hormone family [28]. *NR2F2* participated in the development and formation of liver and adipose tissue, and it was found that a decline of *NR2F2* led to an adipogenesis reduction [29], but Xu et al. (2008) reported that the knockdown of *NR2F2* promoted the expression level of adipocyte marker proteins and fat deposition [30]. These two results were in stark contrast, which may be due to the fact that the differentiation stages of the 3 T3-L1 used in their research were different. Our results was consistent with Xu’s research, castration down-regulated *NR2F2* and ENSMUST00000189966 expression, it was inferred that serum testosterone level can affect their expression, and this effect was confirmed in 3 T3-L1 cell by adding DHT.

## Conclusions

In total, 343 lncRNAs were identified in SAD from both castrated and intact male pigs, and 18 lncRNAs were found to have more than 2-fold differential expression, with *q*-values < 0.05. The pathways analyses suggest that fatty acid synthesis and metabolism was more active in castrated pigs. In the 3 T3-L1 cells, the response of ENSMUST00000189966 and *NR2F2* to differentiation and DHT indicated that lncRNAs with their target genes may play a regulatory role in testosterone deficiency-related fat deposition. We first combined sex hormones, lncRNAs, and genes together; these results provide a novel view on the role of lncRNA in testosterone deficiency-related fat deposition.

## Methods

### Experimental animals

The pigs used in this research were an indigenous Chinese breed, Huainan pigs. This breed was one of the selected superior livestock breeds in Henan province and shows heat-resistance, roughage-resistance, high litter size, and particularly high fat deposition. The pigs used in this research were reared at Henan Xing Rui Agricultural and Animal Husbandry Technology Co., LTD.

Six male Huainan pigs (three pairs of full sibs, all the pigs with similar birth weights) were used in this experiment. On day 35, for each pair of pigs, one was randomly selected to be castrated under anesthesia, and the other one was given a sham operation. All pigs were allowed access to feed and water ad libitum under normal conditions [24], and all efforts were made to minimize suffering.

### Sample collections

All the pigs were slaughtered when their body weight reached around 130 kg (about day 300–315 of age). Before they were slaughtered, their body weight was measured and blood samples were collected from each pig. The blood samples were centrifuged for 15 min at 3000×g at 4 °C, and then serum was collected and stored at −80 °C [31]. The carcasses were eviscerated according to standard commercial procedures, and then the carcass weight and leaf fat weight were obtained. Back fat thickness at the 6–7th rib was measured using a ruler on the left side of the carcass. Subcutaneous adipose tissue samples were collected from the left side of the carcass and were rapidly frozen in liquid nitrogen, then stored at −80 °C until analysis.

### Serum testosterone and TG analysis

The serum testosterone and TG were measured using commercial enzyme-linked immunoassay kits (Beijing North Institute of Biotechnology, Beijing, China; Applygen Technologies, Beijing, China), from the absorption of 450 nm and 550 nm [32], with assay detection range were 0.05 to 20 ng/mL and 0.02 to 2 mM, respectively.

### Histological analysis of subcutaneous adipose tissue

After slaughter, subcutaneous adipose tissue from six pigs were collected and fixed in 10% neutral formalin solution for 24 h, embedded in paraffin blocks, and sectioned to a 6-um thickness. The sections were stained with hematoxylin and eosin (H&E). Then, the slides were viewed and photographed using an Olympus Microscope (Olympus BX51, Tokyo, Japan). The mean adipocyte areas were measure using ImageJ software (NIH, Bethesda). About 50 different fields were captured for each group, 3 fields for each slide, and 5 slides for each pig.

### Intramuscular fat content (IMF) of the LDM and PMM

The IMF of the LDM and PMM was measured using the soxhlet extraction method [33].

### LncRNA library construction and SOLiD sequencing

Total RNA were extracted from subcutaneous adipose samples with TRIzol reagent (Invitrogen, Carlsbad, CA, USA). The RNA integrity, purity, and quality were detected using agarose gel electrophoresis, Nano-Drop ND-2000 spectrophotometer (NanoDrop products, Wilmington, USA), and Agilent 2100 Bioanalyzer (Agilent Technologies, Massy, France), respectively. The concentration of the isolated RNA was measured using a Qubit RNA BR assay for the Qubit 2.0 fluorometer. An RNA Integrity Number (RIN) value of the samples that was greater than eight could be used in sequencing.

The rRNA was removed using an epicentre Ribo-Zero™ kit (Epicentre, Madison, WI, USA) from total RNA and then the RNA was fragmented (150–200 bp) with a fragmentation buffer. The double-strand cDNA was synthesized using SuperScript II reverse transcriptase (Life Techonologies, Saint Aubin, France). Then, cDNA was subjected to end-repair and phosphorylation, and subsequent purification was performed using Agencourt AMPure® XP beads (Beckman Coulter, Villepinte, France). After quantitated by Agilent 2100 Bioanalyzer, the cDNA libraries were sequenced on the Illumina HiSeq 2000.

### Transcriptome assembly

Clean reads was obtained by filtering the adapter and low-quality reads from the raw reads. The TopHat2 software was used to map the clean reads to the porcine reference genome (Sscrofa10.2). Transcriptomes were assembled with Cufflinks supported through Galaxy.

### Pipeline for the identification of lncRNA

The lncRNA was identified by the following steps: (1) Transcripts were removed that were detected in fewer than two experiments. (2) Transcripts with single exon and with the length less than 200 bp were removed. (3) Transcripts with coverage of less than 3 were removed; (4) Transcripts similar to or the same as known porcine small RNAs were removed. (5) Transcripts that did not pass any analyses of Coding Potential Calculator (CPC), Coding-Non-Coding-Index (CNCI), and Pfam software were removed.

### Prediction of target genes

Based on the genome location of the lncRNAs and protein-coding genes, the protein-coding genes that were located upstream and downstream (within 10 kb and 100 kb) of lncRNA were identified as target genes. The gene ontology (GO) terms of the nearest protein-coding genes with highly similar expression patterns were mapped to lncRNAs for enrichment analysis [34].

### Analysis of the relationship between lncRNAs with C/EBPα and miRNAs

The binding sites of C/EBPα in the upstream 2000-bp sequence of 343 lncRNAs were analyzed using MATINSPECTOR 8.0 (Genomatix software GmbH) on its default settings.

LncRNAs may act as targets of miRNAs; miRanda software was used to predict the binding sites of miRNAs in the lncRNA promoter region. We analyzed the relationship between the 343 identified lncRNAs with the 13 promoting adipogenesis miRNAs (let-7、miR-9、miR-15a、miR-17、miR-21、miR-24、miR-30、miR-103、miR-107、miR-125b、miR-204、miR-210、and miR-378) and five depressing adipogenesis miRNAs (miR-27, miR-150, miR-221, miR-222, and miR-326).

### Reverse transcription polymerase chain reaction and real-time PCR

Total RNA was isolated using TRIzol reagent (Invitrogen, Carlsbad, CA, USA) according to the manufacturer’s instructions. First strand cDNA was synthesized using Oligod (T)18 primer and catalyzed by PrimeScript® RT reagent Kit (Perfect Real Time) (TaKaRa, Japan). The primers used in qPCR were shown in Table 2. The reaction contained 10 μL of 2 × SYBR Premix Ex Taq™, 0.4 μL of 50 × ROX Reference Dye II, 0.5 μL of 10 μM forward and reverse primers, 2 μL of template cDNA and ddH_2_O, reaching a final volume of 20 μL. The relative lncRNA expression values were calculated using the 2^-∆∆Ct^ method.

**Table 2:** Information of the primers used in this study

Loci	Primer senquence		Size	Primer Efficiency
	Forward (5′-3′)	Reverse (5′-3′)	(bp)	(mean ± SE)
TCONS_00311264	TGTGGAGGAAACGAGGGT	GAAAGATGCCGCTTGGA	224	0.95 ± 0.01
TCONS_00392681	TATTCTGGGCAGGATTG	GCAGGTCTTTCGAGGTA	160	0.97 ± 0.04
TCONS_01199263	CTCGCTCAACTCAGCATTC	TGGACAAGGGTTAGAAGTAAAT	228	0.84 ± 0.04
TCONS_01743394	TATTGAAATGCGTGAGGCT	CTGGAACGAGCAAGGAGA	187	0.88 ± 0.02
TCONS_00465264	GCTGCGCTCTATTCTGTG	GTATGCCAGGAGGGAGGA	121	0.86 ± 0.02
TCONS_00625772	GGCTTCCGCCTCGTTGAT	CTGGCTGCCTTCGTTTCC	122	0.94 ± 0.06
TCONS_00869992	AGGGAGAAGGAGTGAGACAAG	GGGAAGGCAGATGGACAA	174	0.92 ± 0.03
TCONS_01520466	TTTCTTGGGTAAACATCTGG	GTGACCTTGTTGGGCAGT	158	0.87 ± 0.05
NR2F2	GGACCACATACGGATCTTC	CTGGCTCCTAACGTACTCTT	190	0.94 ± 0.04
ENSMUSG00000100005	GATTAGAGTATCGCATCAGC	CGTGGGTCAGCAAGGTTA	296	0.95 ± 0.02

### Cell culture and differentiation

3T3-L1 cells (Type Culture Collection of the Chinese Academy of Sciences, Shanghai, China) were maintained in the “normal medium”: Dulbecco’s modified Eagle’s medium (DMEM) supplemented with 10% newborn calf serum (NBCS) and antibiotics (penicillin, 100 IU/mL; streptomycin, 100 μg/mL) at 37 °C and 5% CO_2_ in a normal atmosphere incubator. Next, the medium was changed to a “maturation medium I” by adding 0.5 mM IBMX, 1 mM DEX, 5 μg/mL of insulin to the “normal medium” (day 2). After an additional 48 h, the cell culture medium was changed to a “maturation medium II” by adding 5 μg/mL of insulin to the “normal medium” (day 4). Then the medium was changed to “normal medium” until day 14, more than 90% of the adipocyte achieved fully differentiation (indicated by the typical appearance of adipocytes). On day 14, the medium was changed to “normal medium” with or without DHT at various concentrations (0, 10^−9^, and 10^−10^ M) for another 48 h.

### Isolation of RNA from nuclear and cytoplasmic

3T3-L1 cells, cultured with differentiation medium in a T-75 flask, were harvested after which achieved fully differentiation. The harvested cells were washed once by using cold PBS. At 4 °C, these cells were centrifuged at 500×g for 3 min. The nuclear and cytoplasmic RNA were isolated using a PARIS™ kit (Life Technologies, USA). The RNA from nuclear and cytoplasmic was reverse transcribed with the equal amount. The primers for detecting the distribution of U6 and NEAT1 genes were modeled after Zhao et al. (2015) [35].

### Statistical analysis

The results are reported as the mean ± standard error mean (s.e.m.). Data analyses were performed using the statistical R package.

## Additional files

Additional file 1: The sequences of identified lncRNAs in FASTA format. (SEQ 514 kb)

Additional file 2: Table S1. The binding sites of CEBPα at identified lncRNAs. (XLSX 27 kb)

Additional file 3: Table S2. Adipogenesis related miRNAs binding sites at identified lncRNAs. (XLSX 66 kb)

Additional file 4: Figure S1. Comparison of features of porcine lncRNAs and mRNAs. Note: Comparison of the lengths (a), ORF lengths (b) and exon numbers (c) of porcine lncRNAs and mRNAs. (DOCX 302 kb)

Additional file 5: Table S3. The differentially expressed lncRNA between castrated and intact male pigs and their distribution. (XLSX 9 kb)

Additional file 6: Table S4. The differentially expressed mRNA between castrated and intact male pigs and their distribution. (XLSX 222 kb)

Additional file 7: Figure S2. Verification of gene expression analysis by quantitative realtime PCR (qRT-PCR). Note: Individual gene expression ratios were calculated using foldchange generated by RNA-seq and plotted against calculations done for the same gene using qRT-PCR. (DOCX 17 kb)

## Abbreviations

AMPK: AMP-activated protein kinase
C/EBPα: CCAAT/enhancer binding protein alpha
CNCI: Coding-Non-Coding-Index
CPC: Coding Potential Calculator
DHT: Dihydrotestosterone
DMEM: Dulbecco’s modified Eagle’s medium
FPKM: Fragments per kilobase of exons per million fragments mapped
GO: Gene ontology
H&E: Hematoxylin and eosin
LDM: Longissimus dorsi muscle
lincRNAs: Intergenic lncRNAs
lncRNAs: Long non-coding RNAs
NBCS: Newborn calf serum
NR2F2: Nuclear Receptor Subfamily 2 Group F Member 2
ORF: Open reading frame
PMM: Psoas major muscle
PPARγ: Peroxisome proliferator-activated receptor gamma
RIN: RNA Integrity Number
SAD: Subcutaneous adipose tissue
TG: Triglyceride
