# Supplementary figures and images for: Identification and characterization of long non-coding RNAs in subcutaneous adipose tissue from castrated and intact full-sib pair Huainan male pigs
Source: BMC Genomics. 2017 Jul 19;18:542. doi: 10.1186/s12864-017-3907-z (PMC5518130; doi:10.1186/s12864-017-3907-z)

**a**
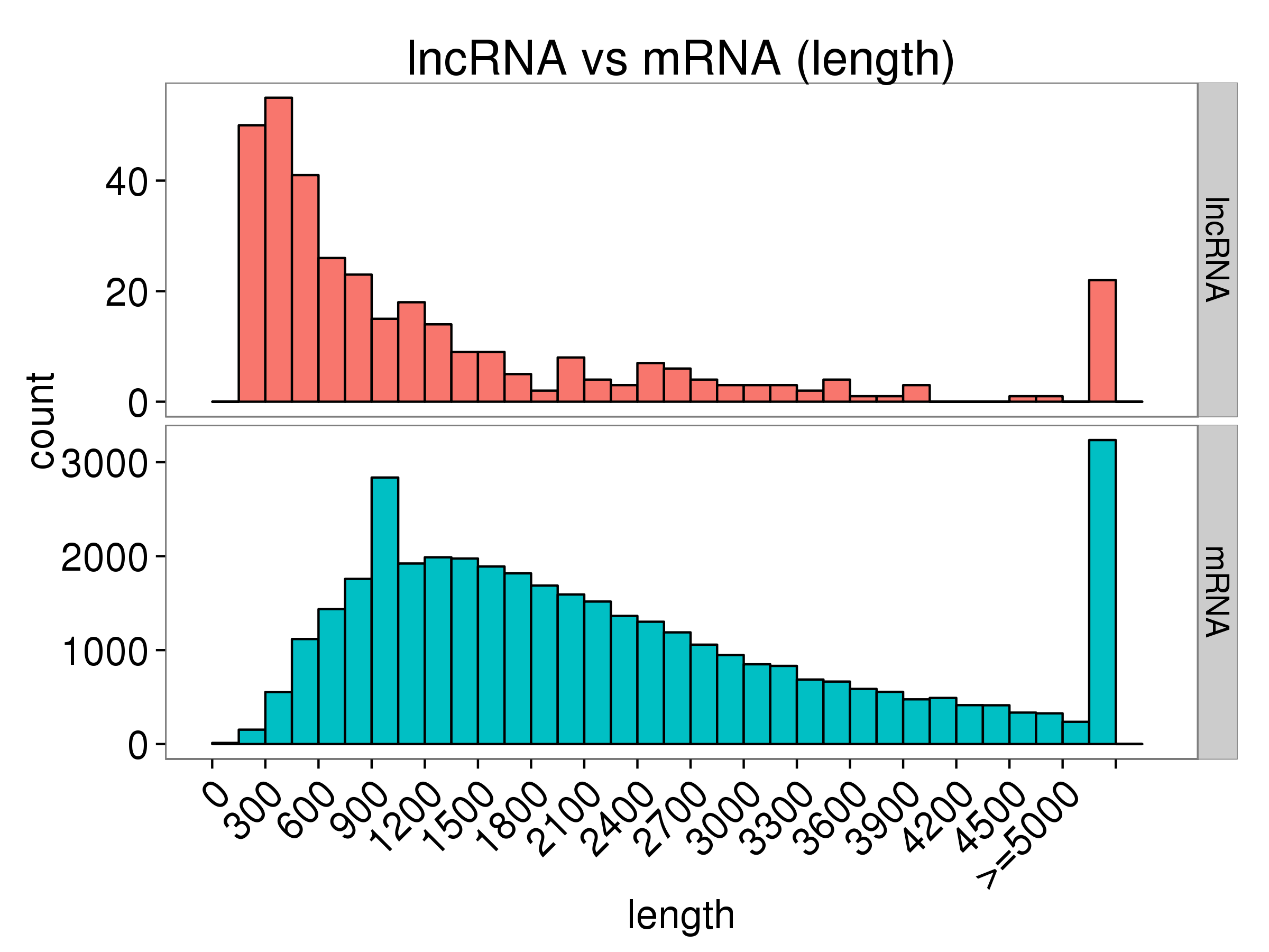


**b**
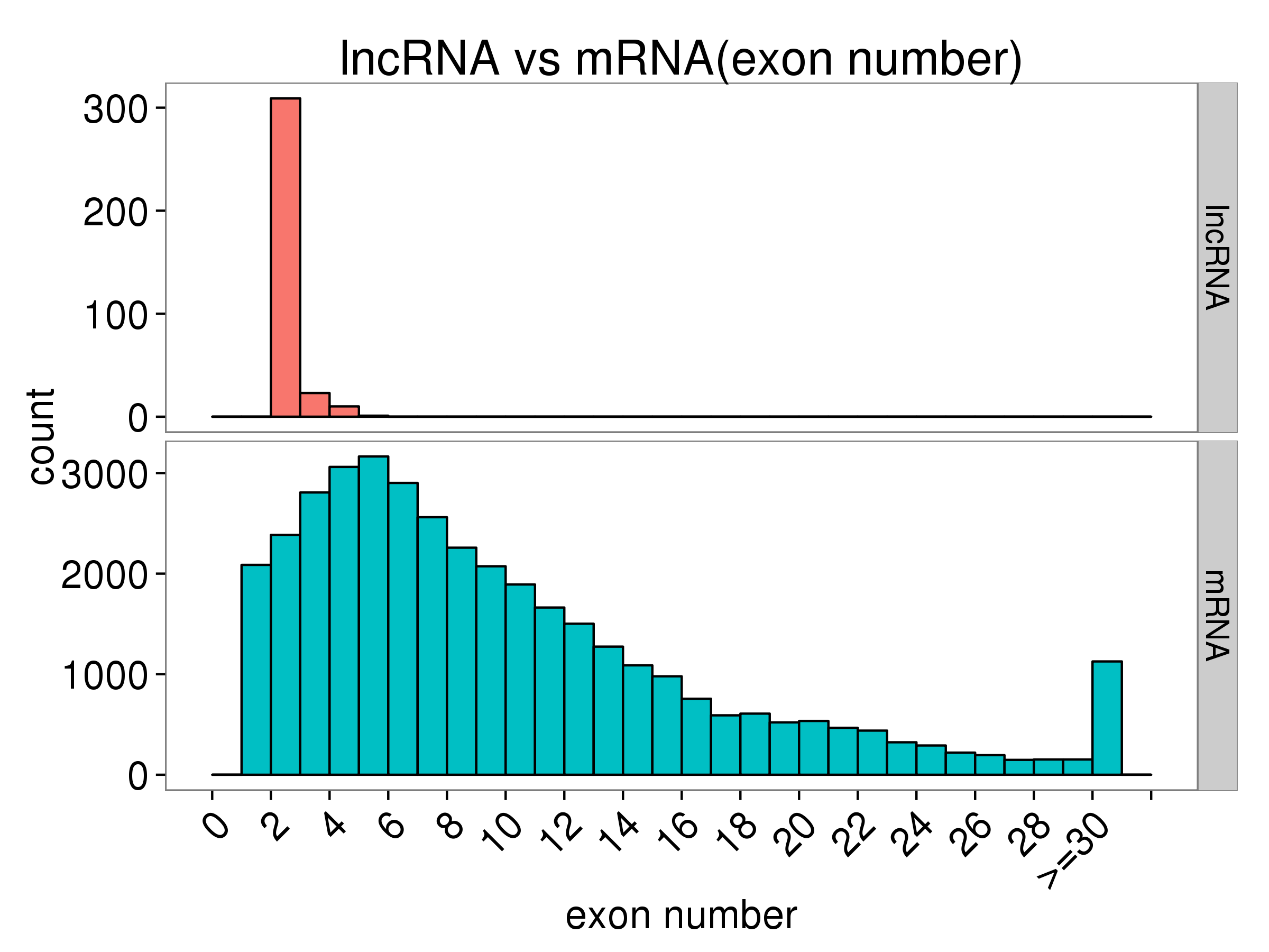


**c**
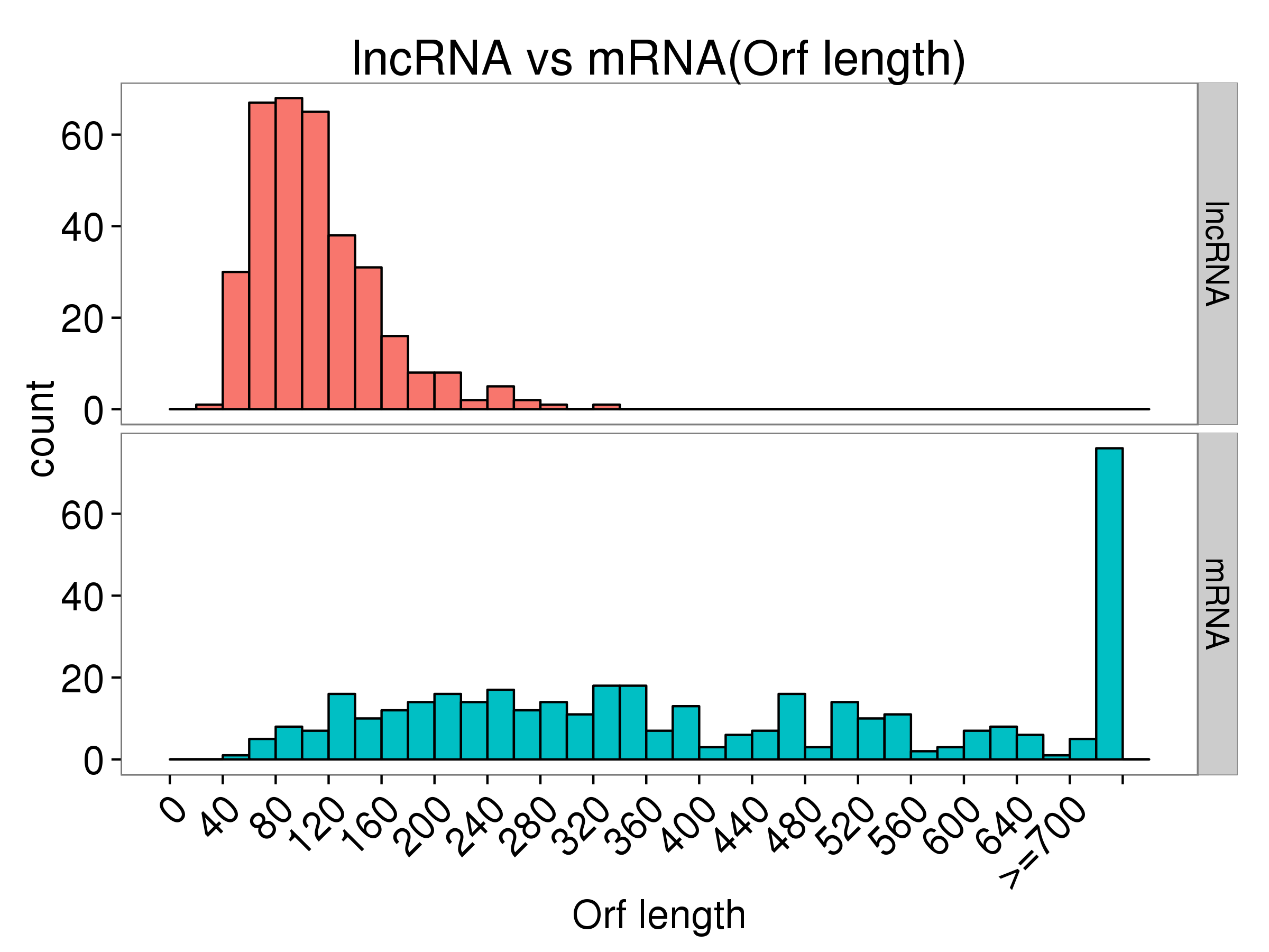

Supplement: Supplementary file 4 — Comparison of features of porcine lncRNAs and mRNAs. Note: Comparison of the lengths (a), ORF lengths (b) and exon numbers (c) of porcine lncRNAs and mRNAs. (DOCX 302 kb) [file 12864_2017_3907_MOESM4_ESM.docx]
