# Supplementary material for: Identification and characterization of long non-coding RNAs in subcutaneous adipose tissue from castrated and intact full-sib pair Huainan male pigs
Source: BMC Genomics. 2017 Jul 19;18:542. doi: 10.1186/s12864-017-3907-z (PMC5518130; doi:10.1186/s12864-017-3907-z)
Supplement: Supplementary file 7 — Verification of gene expression analysis by quantitative realtime PCR (qRT-PCR). Note: Individual gene expression ratios were calculated using foldchange generated by RNA-seq and plotted against calculations done for the same gene using qRT-PCR. (DOCX 17 kb) [file 12864_2017_3907_MOESM7_ESM.docx]

Figure S2. Verification of gene expression analysis by quantitative realtime PCR (qRT-PCR). Note: Individual gene expression ratios were calculated using foldchange generated by RNA-seq and plotted against calculations done for the same gene using qRT-PCR.
